# Regeneration and Single Stage Batch Adsorber Design for Efficient Basic Blue-41 Dye Removal by Porous Clay Heterostructures Prepared from Al13 Montmorillonite and Pillared Derivatives

**DOI:** 10.3390/ma17204948

**Published:** 2024-10-10

**Authors:** Saheed A. Popoola, Hmoud Al Dmour, Rawan Al-Faze, Mohd Gulfam Alam, Souad Rakass, Hicham Oudghiri Hassani, Fethi Kooli

**Affiliations:** 1 Department of Chemistry, Faculty of Science, Islamic University of Madinah, Al-Madinah Al-Munawwarah 42351, Saudi Arabia; gulfam@iu.edu.sa; 2 Department of Physics, Faculty of Science, Mu’tah University, Mu’tah 61710, Jordan; hmoud79@mutah.edu.jo; 3 Department of Chemistry, Faculty of Science, Taibah University, Al-Madinah Al-Munawwarah 41147, Saudi Arabia; rafaze@taibah.edu.sa; 4 Laboratory of Applied Organic Chemistry (LCOA), Chemistry Department, Faculty of Sciences and Techniques, Sidi Mohamed Ben Abdellah University, Imouzzer Road, P.O. Box 2202, Fez 30000, Morocco; rakass_souad@yahoo.fr; 5 Engineering Laboratory of Organometallic, Molecular Materials and Environment (LIMOME), Faculty of Sciences, Chemistry Department, Sidi Mohamed Ben Abdellah University, P.O. Box 1796 (Atlas), Fez 30000, Morocco; oudghiri_hassani_hicham@yahoo.com

**Keywords:** basic blue-41, porous clay heterostructures, pillared clay minerals, mesoporous materials, regeneration, single stage batch design

## Abstract

Porous clay heterostructures are a hybrid precursor between the pillaring process and organoclays. In this study, the organoclay was substituted by an aluminium intercalated species clay or pillared alumina clays. A porous clay heterostructure was successfully achieved from an aluminium intercalated species clay, due to the easy exchange of the aluminium species by the cosurfactant and silica species. However, using alumina pillared clays, the porous clay heterostructures were not formed; the alumina species were strongly attached to clay sheets which made difficult their exchange with cosurfactant molecules. In this case, the silica species were polymerized and decorated the surface of the used materials as indicated by different characterization techniques. The specific surface area of the porous clay heterostructure material reached 880 m^2^/g, and total pore volume of 0.258 cc/g, while the decorated silica alumina pillared clays exhibited lower specific surface area values of 244–440 m^2^/g and total pore volume of 0.315 to 0.157 cc/g. The potential of the synthesized materials was evaluated as a basic blue-41 dye removal agent. Porous clay heterostructure material has a removal capacity of 279 mg/g; while the other materials exhibited lower removal capacities between 75 mg/g and 165 mg/g. The used regeneration method was related to the acidity of the studied materials. The acidity of the materials possessed an impact on the adopted regeneration procedure in this study, the removal efficiency was maintained at 80% of the original performance after three successive regeneration cycles for the porous clay heterostructure. The Langmuir isotherm characteristics were used to propose a single-stage batch design. Porous clay heterostructures with a higher removal capacity resulted in a decrease in the quantities needed to achieve the target removal percentage of the BB-41 dye from an aqueous solution.

## 1. Introduction

One of the greatest challenges before material scientists is to develop material from cheap natural resources and produce chemicals by adopting greener technologies. Among the viable alternatives available for green synthetic methods, clays and clay-based catalysts, in particular, have attracted significant attention due to their high abundance and low cost. Clay minerals are the most common material on earth and are crucial for the development of precious materials. In addition to classical applications, the design of new materials from layered silicates and especially clay continues to grow, and it would be beneficial to figure out how to conceive materials for a specific application [1,2,3]. One of these applications is as a remedial environment in different fields [4].

In recent years, pollution-related issues have become increasingly prevalent and have consistently been a cause of public concern due to society’s demands for freshwater resources and their scarcity [5]. Apart from traditional contaminants, a new and noteworthy category of water pollutants has surfaced. When these pollutants are present in water, even in trace amounts, it can have toxicological effects on both humans and animals who may drink the polluted water [6]. Different techniques were employed to reduce the effect of these contaminants on the quality of water [7]. Each application has its own advantages and disadvantages [8]. Among these methods, adsorption is one of the most effective for removing dyes from wastewater [9]. Clay-based materials have a high stability to release metal ions into an aqueous solution, depending on how they are prepared. This feature, along with the fact that their use is harmless for the environment, makes them ideal for use in water treatment procedures [10].

In the adsorption process, clay-based materials with higher surface areas are favored. To achieve this goal, two different methods for modification were reported: acid activation and pillaring processes [11,12,13]. These days, a lot of attention is focused on acid-activated clays as adsorbents and catalysts for many acid mediating procedures. In the meantime, the pillaring process has become a very promising method since it makes it possible to produce materials (PILCs) with an enhanced surface area, porosity, basal spacing, and good resistance and thermal stability [14]. Pillared clays are a viable option for water treatment operations because of these qualities as well as their affordability and environmental safety [14,15].

A three-step process can be used to obtain PILCs from smectite clay minerals: (a) prepare the pillaring solution containing the pillaring cations (such as Al^3+^, Ga^3+^, Ti^4+^, Zr^4+^, Fe^3+^, and Cr^3+^); (b) intercalate these cations into the clays’ interlayer space, via the exchangeable cations between the clay mineral sheets; and (c) calcine the filtered material under moderate conditions [13]. Due to its well-established chemistry and ease of synthesis in the lab by hydrolysing Al_13_ solution by a base one at particular Al/OH ratios. The polyoxycations of Al_13_ is the most well-documented pillaring agent [15]. In some cases, this type of solution is readily available at large scale.

In early 1992, scientists at the Mobil Corporation discovered novel ordered mesoporous materials and named them MCM-41 (Mobil Composition of Matter No. 41) [16]. The basic idea was the templating of alkylammonium-based surfactants to produce ordered porous silica materials with pore diameters between 1.6 and 10 nm, which are bigger than those of zeolites [17]. When exposed to a certain concentration, the alkylammonium surfactant acts as a structure-directing agent, forming ordered channels. Tetraethyl orthosilicate (TEOS), an inorganic precursor of silica, is thus employed to produce solid walls around the channels through condensation and hydrolysis. A porous silica substance is produced once the template is removed [18,19]. Since then, significant studies on the template-based synthesis to produce silica materials have been made possible by ordered silica based mesoporous materials [19].

The so-called “porous clay heterostructure (PCH)” was first described by Pinnavaia et al. in the mid-1990s as a novel way of synthesizing porous layered silicate using a template approach [20]. In contrast to standard PILCs, the PCH synthesis produces silica-based materials with a uniform pore size distribution; nevertheless, it is restricted to using tetraethyl orthosilicate (TEOS) as a silica source. PCH materials often fill the pore-size gap between mesoporous materials and pillared clays and microporous zeolites, but the process still starts with an organoclay preparation [21,22]. These materials were used in different applications as reported in a recent review [22]. Certain metals must be inserted, either by impregnation or in the PCH silica framework for a particular catalytic application [23,24,25]. This process required additional experimental steps, and it consumed a lot of time. In 2013, Kooli et al. reported that the modification of the starting clay mineral by a cetyl alkyl ammonium surfactant was not necessary and it was substituted by a pre-intercalation of Al_13_ polyoxocations or a zirconium species prior to the synthesis of PCH materials [26,27,28]. This method was able to hit two birds with one stone: first, to reduce the used amount of surfactant reagent; and second, to insert metal cations directly in the silica inserted species in one spot [27,28]. The efficacy of this technique has been demonstrated, as it enables the incorporation of metals into the PCH materials with unique characteristics for catalytic applications such n-heptane hydroisomerization and the elimination of dyes from contaminated water [25,26,27,28,29]. The insertion of aluminium in the silica framework has improved the acidity of the resulting Al-PCH materials [27]. During the regeneration process of adsorbents after removal of basic blue-41 or eosin-Y dyes, the acidity played an important factor using the oxone and cobalt solution [28,29,30]. Since, the pillared clay minerals have an enhanced acidity, so the combination of the pillaring process and synthesis of the PCH could be an efficient adsorbent and easy to be regenerated.

In case of alumina pillared clays, the last step of the pillaring process with Al_13_ polyoxocations consists of calcining the intercalated precursor in the temperature range from 300 to 700 °C. Attempts were made to prepare the derivative PCHs from these calcined materials using the same procedure reported elsewhere. The effect of the calcination temperature of pillared clays prior the synthesis of the PCH was studied. Several techniques, such as XRF, XRD, ^27^Al and ^29^Si solid MAS NMR, TGA, nitrogen adsorption isotherms, and acidity in terms of H^+^ concentration, were used to characterize the materials. Some investigations were carried out to study if the synthetized materials could remove the basic blue-41 dye from artificially polluted water.

The basic blue-41 dye was the interest of team activities due to its wide applications in different fields [31,32,33], and to its dangerous effect on the aquatic environment [33,34]. To optimize the efficiency of these materials, different parameters were investigated separately, including pH (2–10), material dose (0.05–1 g), and initial dye concentration (50–500 mg/L). The experimental data was used to estimate the maximum removal capacity of the different materials. Additionally, a friendly method based on the use of an oxone and cobalt solution has been used to evaluate the regeneration of selected materials for six consecutive cycles to assess their feasibility. This regeneration is crucial for a cost-effective large-scale application. A single batch design was suggested using the theoretical model of basic blue-41 dye removal in the laboratory scale.

## 2. Materials and Characterization

### 2.1. Chemicals and Materials

The reagents and chemicals used are of a scientific grade and were utilized without further purification. The Reheis Chemical Company (Berkeley Heights, NJ, USA) supplied the pillaring agent solution, which is aluminium chlorhydrate hydroxide (Chlorhydrol, 50%). The clay mineral (Ca-montmorillonite, STX-1, with a cation exchange capacity of 92 meq/100 g) was acquired from Purdue University’s Source Clays repository in the United States and identified as Mt clay. For removal investigations, Sigma Aldrich (Merck, Rahway, NJ, USA) has provided tetraethoxide orthosilicate (TEOS), dodecylamine (C_12_amine) surfactant, sodium hydroxide (NaOH), hydrochloric acid (HCl), and basic blue-41 dye (C_20_H_26_N_4_O_6_S_2_ with a colour index number of 11105). Cobalt nitrate salt and oxone were provided by Across Organics (Loughborough, UK).

Unless otherwise specified, all experiments used deionized water.

### 2.2. Preparation of Al_13_ Intercalated Materials and Its Pillared Derivatives

There are two phases in the pillaring process. The pillaring agents intercalate between the layers of host clay during the first phase. The next phase involves calcining these precursors into stable pillars to create the final structure of pillared clay.

Using a cation exchange procedure, 5.2 g of chlorhydrol solution were dissolved into 200 mL of deionized water, then the solution was aged at 80 °C for one hour before 5 g of dry powdered Mt were added. The mixture was stirred for one more hour at 80 °C. After cooling the suspension to room temperature, the sample was filtered, repeatedly cleaned with deionized water, and left to air dry for an entire night. The sample will be identified as Al-IMt, where I stands for the aluminium intercalated species.

To obtain the alumina pillared clays, 5 g of the prepared Al-IMt were treated at different temperatures from 300 to 900 °C in a Carbolite furnace under static air atmosphere at a heating rate of 3 °C for a period of 6 h. The resulting pillared materials will be designated as Al-PMt(X), where X is the calcination temperature and P denotes the pillared state. For instance, Al-PMt(300) refers to pillared Mt clay that was calcined at 300 °C.

### 2.3. Procedure to Prepare PCH Material

The PCH material was synthesized based on previous work [27]. Briefly, Dodecylamine (C_12_H_25_NH_2_) and TEOS were reacted with 1 g of Al-Mt(X) in a molar ratio of about 1/20/150 as the starting materials. After 4 h of stirring at room temperature, the recovered precursor was filtered and air-dried overnight. The sample was labelled as PAl-Mt(X)CH. For example, PAl-Mt(300)CH means that the precursor was prepared from the pillared Al-PMt(300).

In one experiment, the above procedure was implemented without using clay mineral, however. Afterwards, PAl-Mt(X)CH materials were obtained by calcining the PCH precursors for 6 h at 550 °C in air.

### 2.4. Exchange with C_12_amine Solution

The intercalated precursor and its pillared derivative calcined at 500 °C were treated separately only with a pure dodecylamine (C_12_amine) at a molar ratio of clay/C_12_H_25_NH_2_ of approximately 1/20. After 4 h of stirring at room temperature, the resulting product was filtered and air-dried overnight.

### 2.5. Removal Process to Remove BB-41

The experiments were performed in batch using 0.05 g of solid and 50 mL of BB-41 solution of the desired concentration (from 50 mg/L to 500 mg/L). The experimental dilution of the BB-41 dye was derived from a 1000 mg/L dye stock solution. The experiments were conducted by agitating the tubes without controlling the pH of the BB-41 at room temperature in a water shaker operating at 120 rpm overnight. All tests were conducted in duplicate, and the average outputs were reported. After batch removal, the supernatant was examined for the BB-41 concentration after filtration through a 0.45 μm nylon filter. The absorbance of the BB-41 dye in each sample was evaluated by measuring the absorbance at 610 nm (λ_max_). The residual concentration was calculated utilizing a standard calibration curve of known dye concentrations. The percentage removal (R%) and removal capacity (q_e_) of the BB-41 dye were then estimated, respectively [28].

The impact of pH (2.0–11.0) was tested with a 0.1 M HCl or 0.1 M NaOH solutions. The effect of the used solid was performed by varying the mass of the materials between 0.050 g and 1 g.

### 2.6. Regeneration Studies

Regeneration is a key factor affecting the application of removal agents. Following the adsorption process, the saturated sorbent was filtered and then regenerated with a solution of cobalt nitrate and oxone. After filtration, the regenerated solid was collected and then cleaned with distilled water. Fresh new BB-41 (C_i_ = 200 mg/L) was added to the solid, and it was stirred continuously for six hours. A repeat experiment with the identical parameters was conducted for the next cycle [29].

### 2.7. Characterization

The elemental analyses of the materials were performed using a Bruker S4 Explorer instrument. X-ray diffraction patterns of powdered samples were recorded in a Bruker AXS D8Advance X-Ray Diffractometer (Karlsruhe, Germany) using Ni filtered CuKα radiation (λ = 1.5406 Å) in the range 2θ from 1.5 to 50. TGA curves of dried samples were analyzed by TA instrument-model 9610 ((New Castle, DE, USA) under atmospheric air, with a heating rate of 10 mL/min from room temperature to 900 °C. Solid State MAS NMR experiments were carried out over a Bruker 400 spectrometer (Karlsruhe, Germany) at a resonance frequency of 78 MHz. For all measurements a standard 4 mm double bearing Bruker MAS probe was used, more details are reported elsewhere [28]. The determination of surface area and pore volume for the materials were obtained simultaneously at liquid nitrogen temperature with a Quantachrome model A6 instrument analyzer (Boynton Beach, FL, USA) followed by degassing at 300 °C overnight. The total acidity was calculated using a thermogravimetric analysis (TG) of the materials following cyclohexylamine adsorption. The adsoprtion of cyclohexylamine was performed by adding 0.2 g of solid to 20 mL of cyclohexylamine in a covered glass Petri dish at room temperature overnight, followed by heating at 80 °C for 4 hrs to remove the physisorbed probe molecule, then kept in a dessicator. The mass loss in the range of 200–420 °C under a nitrogen atmosphere for each material was used to compute the acidity in terms of mmol of cyclohexylamine adsorbed. The concentrations at equilibrium of BB-41 at the absorbance at maximum wavelength (λ_max_ = 610 nm) were estimated from the calibration curves using a UV-Visible spectrophotometer Varian (carry 100, Varian, Victoria, Australia). The pH of point zero charge (pH_PZC_) of selected materials was estimated by the pH drift method.

## 3. Results and Discussion

### 3.1. XRF Analysis

The starting clay mineral, pillared derivatives, and PCH materials were analyzed by XRF; the results are presented in Table 1. The starting clay had reasonable amounts of CaO, in addition to traces of K_2_O. During intercalation of the Al_13_ species, the percentage of CaO decreased indicating that the pristine clay exchanges Ca^2+^ cations to an aluminium species, followed by an increase in Al_2_O_3_ content. K_2_O makes strong connections with clay, which likely explains why K_2_O decreased less than CaO [35].

As a result, the pillaring agent intercalated into the clay structure, indicating that the intercalation procedure was successful. The XRF of pillared clay minerals (after calcination at different temperatures) indicated that there was a slight variation in composition for SiO_2_ and Al_2_O_3_. The Al_2_O_3_ content varied from 22% to 23%. The dehydroxylation of the Al_13_ species and the clay mineral sheets were linked to the small change in the Al_2_O_3_ content.

After reaction with TEOS and the amine template, the chemical composition was significantly altered. The SiO_2_ percentage increased from 57% to 80% and the Al_2_O_3_ decreased from 22% to 5.4%. The addition of a silica source during the PCH preparation resulted in an increase in SiO_2_ [36], and to the replacement of the Al_13_ species with C_12_amine, respectively [26]. Indeed, the XRF analysis of the Al-IMt exchanged with pure C_12_amine revealed a decrease of the Al_2_O_3_ content without a variation of the SiO_2_ percentage content.

The percentage of Al_2_O_3_ in the PCH materials made from pillared Al-PMt(X) clays was slightly affected by the only apparent reduction in SiO_2_ content from 6% to 4% (Table 1). The strong chemical bonds formed during the calcination process between the clay sheets and the alumina species render them difficult to be substituted with C_12_amine [27]. In this instance, the pillared clays’ exterior surface could be decorated by the silica species. The Al-PMt(500) XRF data exchanged with only C_12_amine demonstrated a minor change in the material’s composition.

### 3.2. Powder XRD Data

**Figure 1:**
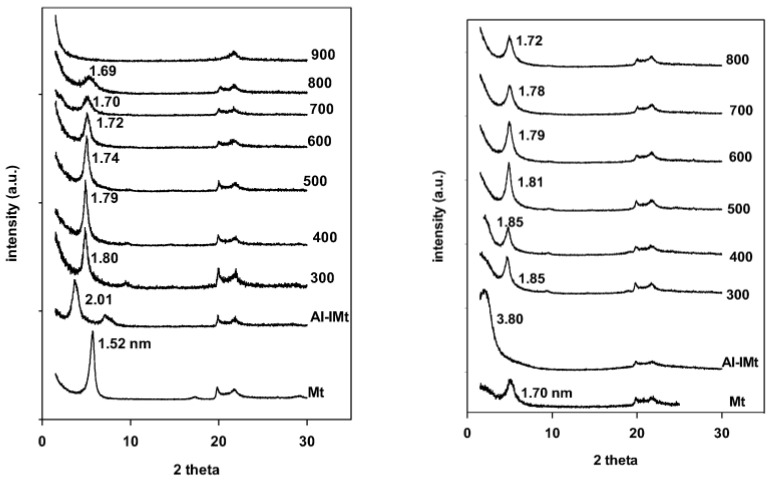
(**left**) PXRD patterns of raw clay, intercalated with the Al_13_ species and calcined at different temperatures; (**right**) after reaction with C_12_amine and TEOS, then calcined at 550 °C.

The PXRD patterns of the starting Mt and its Al-PMt(X) are presented in Figure 1 (left). The parent clay’s PXRD pattern showed a reflection at 5.2 (2θ) value, which corresponded to a 1.52 nm basal spacing (d001). The latter provides evidence that the parent clay mineral is in the Ca-montmorillonite form, with Ca^2+^ acting as the most exchangeable cation [36]. Compared to clay minerals substituted with monovalent cations (e.g., Na+), the clay minerals substituted with divalent cations have higher d001 values because the hydration layer is larger [37]. The PXRD data of the Al-IMt precursor showed that the 001 reflection shifted to a small angle of 3.42 (2θ), while the basal spacing increased to 2.02 nm after reaction with the chlorhydrol solution. This shift is attributed to the successful exchange of Ca2+ cations with the polymeric aluminium species. The Al-IMt precursor exhibited an interlayer distance of 1.13 nm, with a 0.96 nm thickness of the Mt layers. This value was somewhat comparable to the size of Al13 polyoxocations [38]. The basal spacing decreased when the Al-IMt precursor was calcined in the 1.80–1.70 nm range. The dehydroxylation of pillaring species up to 500 °C was attributed to this decrease. The clay sheets underwent dehydroxylation at temperatures higher than 500 °C, which resulted in a progressive reduction in the 001 reflection’s intensity [39]. The layers’ structure had totally collapsed at 900 °C, and there was no longer any evidence of the 001 reflection [39].

The PXRD of the samples after reaction with C_12_amine and TEOS indicated that one main reflection was observed for the PAl-IMtCH with a basal distance of 3.80 nm. This expansion demonstrated the successful synthesis of the PCH precursors and the co-intercalation of silica species and C_12_amine between the clay layers (Figure 1 right). Multiple reflections were absent from the PXRD pattern, which is in line with reported data on conventional PCHs [40,41]. According to published data, the PXRD patterns of PCHs exhibited a basal spacing of 1.98 nm [42] or showed very little change following the reaction with TEOS and C_12_amine [43]. In other cases, some PCH materials did not at all exhibit a reflection at a lower angle and was related to the formation of an exfoliated structure [44].

When the pillared montmorillonite materials (Al-PMt(X)) were reacted with C_12_amine and TEOS, the PXRD patterns of the resulted samples indicated that there was no significant change in the basal spacing, and they were similar to the used Al-PMt ones. This fact suggested that the PCH preparation was unsuccessful [27], with a slight increase in crystallinity. Similar findings from earlier research showed that the pillaring species were difficult to exchange with the C_12_amine because they were firmly bonded to the clay sheets upon calcination. In fact, there was no rise in the basal spacing seen in the interaction between Al-PMt(500) and pure C_12_amine [45]. The PCH preparation was not achieved using pure Mt without pre-expansion of the interlayer spacing, and a phase of d_001_ of 1.71 nm was obtained (Figure 1 right), demonstrating the necessity of expanding the interlayer spacing as a first step prior to the synthesis of the PCH precursors [26].

According to Barakan and Aghazadeh (2020), it may be possible to prepare the PCH from Al and Fe pillared clays by employing an ultrasonic technique to disintegrate the pillaring species before the mixture reacts with a C_12_amine and TEOS combination [46]. This approach was effective in facilitating the microwave synthesis of the PCH [46]. The pillared material in this study was attempted to be treated in an ultrasonic bath for 30 min, but the synthesis of the PCH material was not successfully accomplished. The ultrasonic bath’s power may be related to this disagreement. Al and Fe pillars are broken by ultrasonication, and the varying hydrolysis rate of the entrapped silica source is accelerated by microwave irradiation [46].

After calcination of the PAl-IMtCH precursor at 550 °C for 6 h in an air atmosphere, the PXRD pattern indicated that there was a slight shift of the basal spacing to a higher angle, corresponding to a basal spacing of 3.63 nm, due to the release of the C_12_amine surfactant and the dehydroxylation of the polymerized silica species inserted in the PCH material. This shift was reported for similar materials [26,41,45]. However, for the materials prepared from pillared Al-PMt(X) there was no shift in the position of the basal spacing of the pillared clays that reflected the stability of the pillared clays at 550 °C, with a slight increase in crystallinity. The PXRD pattern of the synthesized sample using pure Mt clay shifted to a higher angle with a d_001_ of 1.14 nm.

Using the Debye–Scherrer equation, the crystallite size of the starting Mt is about 20.2 nm; after pillaring the size depended on the calcination temperature. The size increased to an average of 25.3 nm for the sample calcined between 300 and 500 °C, then decreased to 20 nm at temperature range from 600 to 700 °C. At 800 °C, a value of 14.4 nm was obtained. However, after reaction with C_12_amine and TEOS, followed by calcination at 550 °C, the crystallite size of the resulting products did not change, with an average value of 25.7 nm for materials prepared from pillared clay calcined at temperatures in the range from 300 to 500 °C; however, it increased to 25.7 nm for samples prepared from pillared clay calcined at temperatures in the range from 600 to 800 °C.

**Figure 2:**
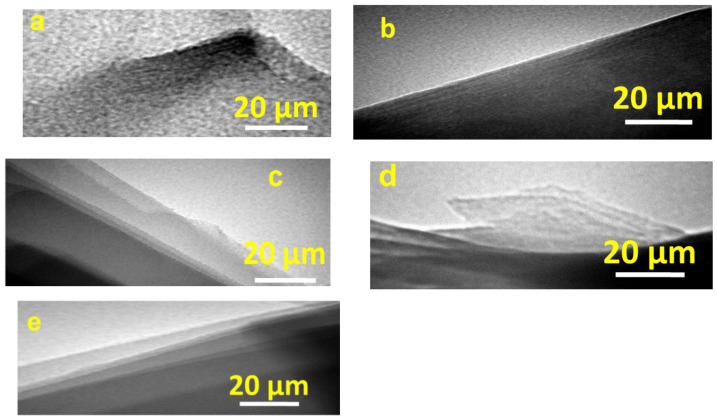
TEM micrographs of (**a**) raw Mt, (**b**) intercalated with the Al13 species (Al-IMt), (**c**) after calcination at 500 °C, (**d**) PAl-MtCH, and (**e**) PAl-Mt500CH.

According to the TEM trials, the parent Mt clay was made up of layers with curved edges (Figure 2a). The layered feature of the Al-Mt intercalated precursor was retained, albeit with some expansion, upon intercalation of the alumina species (Figure 2b). Upon calcination of the intercalated precursor at 500 °C (Figure 2c), the layered structure was maintained. After reaction with C_12_amine and TEOS, the distance between the layers kept on swell for the PAl-IMtCH (Figure 2d), and a slight change occurred for the resulting material pillared clay mineral (Figure 2e).

### 3.3. ^29^Si and ^27^Al MAS-NMR Data

The solid magic-angle spinning (MAS) NMR technique was employed to identify the several Al species found in the PCH materials as well as the surrounding Si environments.

**Figure 3:**
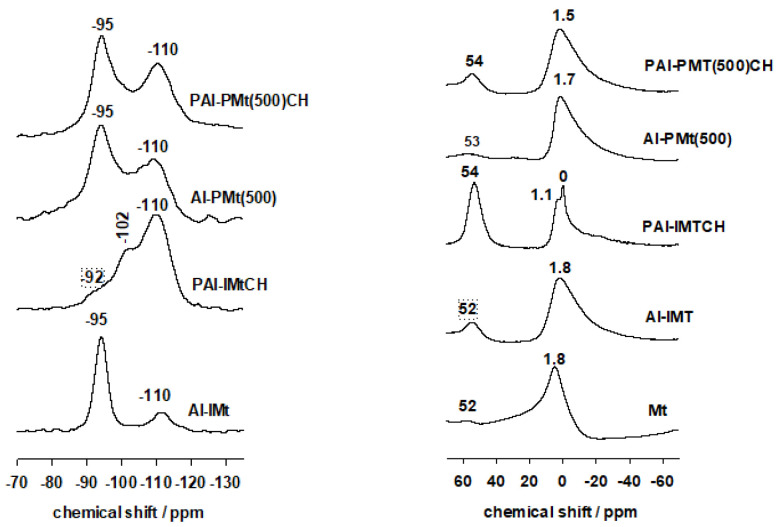
(**left**) ^29^Si MAS NMR and (**right**) ^27^Al MAS NMR of the different materials.

The parent Mt exhibited an intense resonance peak at −95 ppm, attributed to Q^3^ Si atoms in the tetrahedral clay layer, which are connected to three other Si atoms of oxygen and one Al or Mg atoms in the octahedral layer [26,47]. A broad peak was also observed at −111 ppm, which was associated with silica impurities in the core clay. Similar data have been published for various clay minerals. [26,45,47]. The intercalation of Al_13_ species in the parent Mt did not change the characteristic of the ^29^Si MAS-NMR spectrum (Figure 3 left), indicating that the basic blueprint of the clay sheets was preserved. The increased calcination temperatures had no effect on the short-range order in the clay layers, with an intense resonance maximum at −95 ppm and broad one at −110 ppm [30].

The Al-PCH precursor showed distinct features (Figure 3, left) with a high resonance signal at −110 ppm and a broad one at −102 ppm. The polycondensation of silicon species in the interlayer gallery of the obtained precursor was confirmed by the absence of the typical resonance peak of TEOS at −81.7 ppm [48]. The peaks, located at −110 and −100 ppm, were assigned to Q^4^ Si species Si(SiO_4_) and Q^3^ Si species (HO) (SiO_3_) of the polymerized TEOS, respectively. In addition to the previously reported resonance at −95 ppm, which was connected to clay layers, the bands’ positions were unaffected by the presence of Al because of their large overlapping. Comparable findings have been published for the PCH precursors made using various procedures [26,39,49].

The dehydration and condensation of the silica species between the clay layers proceeded when the precursors were calcined at 550 °C, and the ^29^Si MAS NMR spectrum consisted primarily of the Q^4^ species [26,40] (Figure 3 left). The resonance peak of the Q^4^ Si species broadened and shifted to −110 ppm. Q^3^ of silica mesoporous was still detected at −102 ppm. However, the Q^3^ Si peak of the clay layers shifted from −95 ppm to −92 ppm and became part of the silica species’ strong peaks. The results demonstrated that while the structure of PAl-IMtCH was maintained, the inserted silica species incurred some modifications.

^29^Si MAS-NMR of the material formed from pillared Al-PMt(500) (Figure 3 left) had a feature comparable to the original material and supported the PXRD data, where no PCH material was formed. Due to the presence of an amorphous silica phase on the outside surface of the Al-PMt(500) material, there was a little enhancing of the intensity associated with the Q^4^ species (Figure 3 left). Comparable results were published using the pillared clays made of alumina or zirconia to prepare possible PCH materials [26,27,28].

The chemical shift of ^27^Al depends strongly on the coordination of the aluminium atom, They are typically 0–5 ppm for an octahedral environment (or 6-fold coordination, Al^VI^) and 50–60 ppm for tetrahedrally coordinated Al (or 4-fold coordination, Al^IV^) referenced to the resonance frequency of Al in Al(NO_3_)_3_ and dissolved in water as 0 ppm) [47]. ^27^Al MAS NMR spectra give the qualitative information on the presence of different coordination of aluminium. It is clearly seen that the raw Mt contains a higher content of octahedrally coordinated Al with a strong peak at 1.8 ppm, in addition to a small amount of tetrahedrally coordinated Al associated with a broad peak at 52 ppm [26,39] (Figure 3 right). The aluminium pillaring species produced an additional peak between 60 and 70 ppm, associated to tetrahedral aluminium (Figure 3 right). However, after intercalation of the Al_13_ species, there was no discernible difference between Mt and Al-IMt [29,38]. Due to the pillar species’ interaction with the clay sheets, the pillared clay (Al-PMt(x)) displayed the tetrahedral peak with a shift of the octahedral one at 1.4 ppm [50,51,52] (Figure 3 right).

The PAl-IMtCH calcined at 550 °C displayed distinct characteristics, showing a significant increase in resonance at 54 ppm associated with tetrahedral Al^IV^, and two resonance peaks related to Al^VI^ at 1.1 and 0 ppm. The latter two peaks may be attributed to the Al^VI^ in the clay layers and an additional Al^IV^ present in different environments, such as within the intercalated silica species [26,45] (Figure 3 right). By contrast, the PAl-PMt(500)CH material exhibited a comparable resonance feature to that of the parent Al-PMt(500) clay, suggesting no alteration of the Al environment, and the absence of porous clay heterostructure material [45], which is consistent with the XRF and PXRD data (Figure 3 right).

### 3.4. Thermogravimetric Data

**Figure 4:**
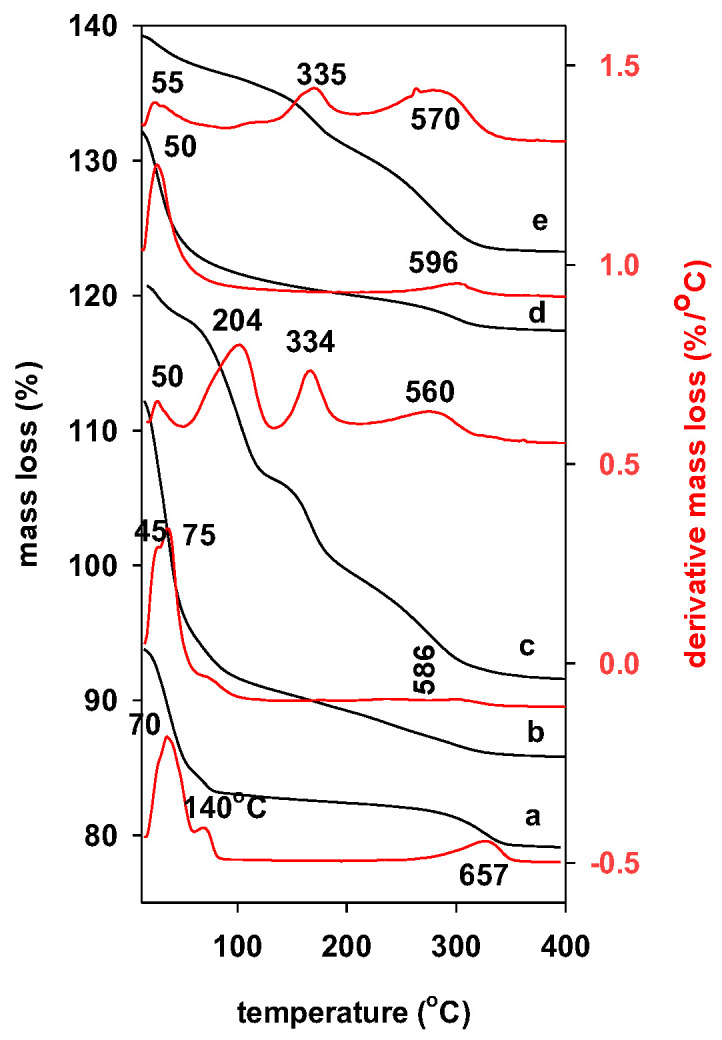
TGA (black) and DTG (red) features of the different materials: (**a**) Mt, (**b**) Al-IMt, (**c**) PAl-IMtCH, (**d**) PAl-Mt(500), and (**e**) derived PAl-Mt(500)CH.

The aim of the thermogravimetric study was to prove the presence of the pillaring species in the intercalated Al-IMT precursor and surfactants in the PCH materials, as well as to identify the temperature thresholds at which the materials retain their stability. The TGA profile of parent clay depicted in Figure 4a indicated a 12% mass loss between 25 °C and 200 °C. This mass loss was associated with the removal of water molecules and physically adsorbed water that are bonded to the exchangeable cations in the interlayer, rising to two DTG peaks at maximum temperatures of 70 °C and 140 °C, respectively [26] (Figure 4a). The additional mass loss of 4.5% between 550 °C and 800 °C was associated to the dehydroxylation of the clay sheets with a maximum temperature loss of 657 °C in the DTG curve.

The intercalated Al-IMt precursor’s feature was comparable to that of Mt with a mass loss of 17% in the range from room temperature to 230 ° C (Figure 4b). This was explained by the dehydroxylation of hydroxy Al_13_ oligomers, the elimination of water from the surface, and pores [52].

The associated DTG curve showed that there were two significant peaks: one at 45 °C and the other at 75 °C with a shoulder. Once the hydroxy Al_13_ oligomers were substituted for the Ca^2+^ cations, the magnitude of the DTG peak (at 140 °C) was reduced. Above 240 °C, a continual mass loss was noted, and a new deflection was noted at 460 °C, which was caused by the conversion of metal hydroxide into metal oxide pillars [51,52]. There isn’t any mass loss beyond 500 °C, which suggests the stability of the Al-IMt precursor, with a shift of the DTG peak to a lower temperature of 597 °C [26,51] (Figure 4c). The insertion of the Al_13_ cations was achieved, as evidenced by the total mass of 21.5% for the intercalated precursor from the 25 to 800 °C range, that exceeded the total mass of the parent clay mineral (16.5%).

Figure 4d displays the TGA and DTG profiles of the PAl-IMtCH precursor (uncalcined) porous clay heterostructure. These profiles differed from the Al-IMt precursor. A significant reduction in mass loss was noted between 25 and 140 °C, ranging from 17% to 4.5%. This suggests that the C_12_amine is exchanging Al_13_ cations, and the surface was changed to hydrophobic [26] (Figure 4c). The two DTG maxima at 204 °C and 334 °C are connected to the mass loss of 20% between 150 °C and 300 °C that is caused by the surfactant C_12_amine elimination in two stages [45,53]. The total elimination of carbon species, the dehydroxylation of silica species, and the dehydration of clay sheets led to an extra DTG peak at 560 °C, that is linked to the continuing mass loss of 8% above 500 °C (Figure 4c). The additional stability of these materials is indicated by the porous clay heterostructure. According to published research, they exhibited greater thermal stability than pillared clays [53].

Only two different mass loss stages were seen in the pillared Al-PMt(500), and these were associated with the dehydroxylation of the clay sheets and physisorbed water, correlated with DTG peaks at 53 and 596 °C, respectively (Figure 4d). About 12% of the entire mass was lost, which is significantly less than the parent precursor (Al-IMT) [54].

The precursor PAl-PMt(500)CH displayed a different curve than the PAl-IMtCH, with an additional mass loss of 6% resulting to the elimination of C_12_amine molecules and associated to a DTG peak of 335 °C, in the 130–420 °C range. Qualitatively, the intensity of the DTG peak associated with physisorbed water at 50 °C was low [44] (Figure 4e). This might be attributed to the hydrophobic nature of the resultant sample, which is caused by the presence of C_12_amine on the pillared clay’s surface. These data showed that synthesis of the PCH precursor was not achieved and confirmed the PXRD findings.

### 3.5. Textural Properties

**Figure 5:**
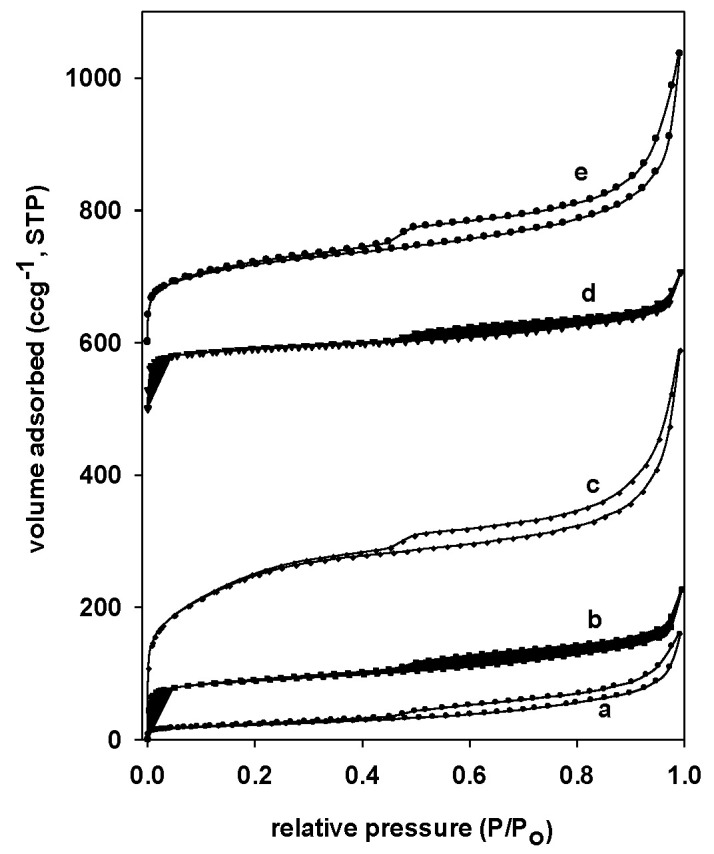
N_2_ adsorption-desorption isotherms of different materials: (**a**) Mt, (**b**) Al-IMt, (**c**) PAlMtCH, (**d**) Al-PMt(500), and (**e**) PMt(500)CH.

The Nitrogen isotherm of Mt clay calcined at 500 °C showed a significant hysteresis loop, a type IV feature of non-porous materials (Figure 5a) [26]. In the meantime, because of the increased interlayer spacing during the pillaring process, the nitrogen adsorption desorption isotherms of the intercalated and pillared materials showed a type I isotherm at lower relative pressures (P/Po) and a type IV isotherm at higher values of P/Po (with an increase of the adsorbed volume at lower relative pressures is observed compared to Mt) (Figure 5b,d) [45]. The hysteresis had a type IV shape, which is associated with the development of mesopores between interlayers. The isotherms’ shapes were comparable when the calcination temperature was raised to 800 °C, because of the partial breakdown of the layered structure and the shrinkage of the interlayer spacing at higher temperatures, the adsorbed values at low relative pressures were decreased [45]. Indeed, PXRD results showed that, for the sample calcined at 900 °C, the form of the isotherm altered to no porous materials because the layered structure was completely destroyed. Reports pertaining to other pillared materials were comparable [27,45].

Notable change in the form of the isotherm was noticed for PAl-MtCH material made from the Al-IMt precursor and calcined at 550 °C. The layout of the adsorption isotherm differed from that of the used precursor (Figure 5c). The only isotherm detected was type IV, which suggests increased porosity and a hysteresis loop characteristic of open cylindrical pores at both sides. It was similar to those reported for typical PCH materials [20,21,25,36]. The isotherm showed a notable rise in nitrogen adsorbed at low to medium relative pressures, suggesting the existence of small mesopores and supermicropores, as well as the ease with which nitrogen molecules might access the micropores [20,40].

The nitrogen isotherms for the PCH made from pillared alumina samples, however, resembled the ones of pillared clays in terms of shape (an example of an isotherm combining types I and IV isotherms is depicted in Figure 5e). The hysteresis loop suggested that the capillary condensation and potential modification of the pore morphologies between the particles were the causes of the observed rise in the amount of nitrogen adsorbed at relatively high pressures of 0.95 [45].

Table 2 lists the textural characteristics of the used samples and their PCH counterparts. The parent clay’s average pore diameter (A.P.D.) is around 5.8 nm, and its total pore volume (T.P.V.) is 0.051 cc/g. Its BET surface area (S_BET_) is 90 m^2^/g [26]. The value of S_BET_ is higher than the values reported for other clay minerals ranging from 19 m^2^/g to 70 m^2^/g [40,55,56,57,58]. The values depended on the origin of the clay mineral and the types of cations located between the layers. The S_BET_ and T.P.V. enhanced after the insertion of the Al_13_ species (Al-IMt) to 318 m^2^/g and 0.262 cc/g, respectively, with some contribution to the micropore volume, as reported in Table 2; a decrease of A.P.D. to 3.23 nm was also noted [38,44]. The calcination of the Al-IMt precursor at temperatures below 600 °C resulted in the formation of pillared materials, with a reduction of the textural properties. At 500 °C, the S_BET_ and T.P.V. reached 281 m^2^/g and 0.237 cc/g, respectively. These values continued to decrease for temperatures in the range from 600 °C to 800 °C and reached values of 165 m^2^/g and 0.023 cc/g, respectively. During calcination at 900 °C, the material showed a substantial reduction to 30 m^2^/g, which was attributed to the total collapse of the layered structure [45,59]. Overall, the pillared materials exhibited a mesoporous character with an A.P.D. of 3.35 nm, with some contribution of micropore volume to the T.P.V. (see Table 2).

The obtained PCH precursors were calcined at 550 °C before the measurement of the textural properties. Concerning the PCH material prepared from the Al-IMt precursor, Table 2 indicates a significant improvement of the S_BET_ and T.P.V. values to 880 m^2^/g and 0.851 cc/g, respectively, with a considerable reduction of micropore volume. The material’s A.P.D. value of 3.82 nm indicates that the material has retained its mesoporous character. The value of the S_BET_ was comparable to the published values of zirconium PCHs and conventional PCHs [27,54], and higher than other PCHs prepared from clay minerals with different origins and types [36,40,41,42,43,53,54,55,56,58]. Thus, the textural properties depend strongly on many factors, and one must be careful when comparing the obtained data. The S_BET_ of the “PCH” materials obtained from pillared Al-PMt(X) samples, ranged from 244 to 453 m^2^/g (Table 2). This value was higher than that of the original pillared clays but significantly lower than that of the PAl-IMtCH (880 m^2^/g). According to ^29^Si MAS NMR (see above), the reaction of C_12_amine and TEOS yielded to an amorphous silica accompanied by an increase of the surface area. The decrease in the micropore volume and the A.P.D. values showed that the amorphous phase could be blocked in some extended pillared clays’ pores. Comparable data was also given for the PCH materials derived from the intercalated precursor zirconium and its pillared derivatives [27].

### 3.6. Acidity Characterization

The materials’ acidity was determined by using cyclohexylamine as a probe molecule and the presumption that each base molecule interacts with one acid site [45]. The data are summarized in Table 3. The parent Mt after 500 °C calcination displayed an acidity of 0.51 mmol H^+^/g, and an enhancement of the acidity is followed by the pillaring process [60]. In fact, at a calcination temperature of 300 °C, the pillared Al-PMt(300) possessed an acidity of 0.71 mmol of H^+^/g of clay. This value continues to decrease at calcination temperatures from 300 °C to 800 °C, reaching a value of 0.23 mmol of H^+^/g of clay for pillared material calcined at 800 °C. The pillaring species’ destruction led to a drop in the number of acid sites, which in turn caused the acidity to decrease [39,45]. At a higher temperature of 900 °C, no acidic material was formed, since the pillared material was completely destroyed. These results closely matched those obtained in studies on alumina-pillared clays [44].

With a concentration of 0.60 mmol of H^+^/g of clay, the acidity of the PAl-IMtCH material was two times higher than that of its original precursor (Al-IMt). This value exceeds that stated for the PCH materials produced via conventional processes [61,62,63]. As reported above, the porous clay heterostructures were not obtained using pillared alumina clays. The prepared materials using Al-PMt(300) and Al-PMt(400) exhibited a higher acidity of 0.73 and 0.62, respectively. This is due to the higher acidity of the original pillared clays. The average value of the PAl-Mt(X)CHs materials was approximately 0.59 mmol of H^+^/g of clay for pillared clay calcined in the range from 500 to 800 °C. In this case, amorphous silica that decorates the surface may interact with the clay sheets, leading to a possible addition to the acidity values as compared to pure pillared clays.

### 3.7. Influence of BB-41 Removal Parameters

The ability of solid’s unit mass to remove a fixed percentage of solute or a given proportion of solute from the solution to a given mass is known as the removal efficiency.

In this study, all the materials are calcined at 550 °C before use, unless otherwise stated.

#### 3.7.1. Effect of the Dose

**Figure 6:**
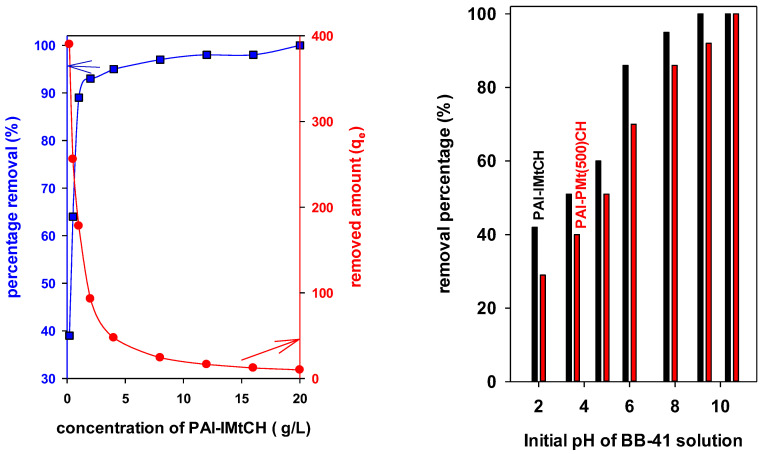
Effect of on the removal properties of BB-41 dye, (**left**) PAl-IMtCH used mass and (**right**) initial BB-41 pH solution.

The effect of the PAl-IMtCH dosage ranging from 0.2 g/L to 20 g/L on BB-41 removal properties is depicted in Figure 6 (left). The increase in the PAl-IMtCH dosage from 0.2 g/L to 8.00 g/L caused an improvement from 39 to 98% in the dye removal percentage (R%). However, the removal capacity (q_e_) was reduced from 390 mg/g to 178 mg/g. A new increase of dose concentration from 8 g/L to 20 g/L slightly affected the dye R% from 98% to 100%; but, the removal capacity continued to decrease from 93 mg/g to 10 mg/g (Figure 6 left).

In the case of the PAl-PMt(500)CH sample, the increase in solid dosage from 0.2 g/L to 8 g/L caused a rise from 23% to 86% in the dye removal percentage (R%). However, the removal capacity (q_e_) decreased from 232 to 21 mg/g. A new increase from 12 g/L to 20 g/L caused an effect on the dye removal percentage from 93% to 98%; but, the removal capacity continued to decrease from 15.5 m/g to 10 mg/g. The improvement in the removal of BB-41 can be explained as follows: since more removal sites are provided by the increased material dosage, more dye then gets eliminated from the solution. Comparable data were described for the PCH materials and some layered silicates [28,29,63,64,65].

Of the two removal solids used in this study, the PAl-IMtCH exhibits greater elimination at all levels of the adsorbent dosing than the PAl-PMtCH because the specific surface area of (880 m^2^/g^−1^) is higher than that of PAl-PMtCH (346 m^2^/g^−1^). Aiming to obtain suitable values of R% and q_e_ for both materials, the 1 g/L adsorbent mass was selected for all removal studies.

#### 3.7.2. Effect of Initial pH BB-41 Solution

The initial pH values of BB-41 solutions ranged from 2 to 11 by the addition of drops of NaOH (0.1 M) or HCl(0.1 M) solutions and the removal tests were performed at an initial concentration of 200 mg/L using 0.050 g of solid materials. As shown from Figure 6 (right), the tests revealed the enhanced removal efficiency (R%) of BB-41 from acidic to basic pH on both the PAl-IMtCH and PAl-PMtCH materials. Using PAl-IMtCH, the dye removal enhanced from 42% to 95%, while the noticeable increase in dye removal has been observed up to pH 8 [28]. The PAl-PMt(500)CH exhibited the same behavior; however, the maximum removal percentage of 93% was achieved at pH 10. At pH values above 10, the strong alkaline environment destroys the BB-41 molecules, resulting in the formation of a brown precipitate [28]. We were unable to evaluate the supernatant as a result. A point zero charge (PZC) and the molecular structure of BB-41 can be used to explain variations in the removal behavior of BB-41 molecules on the PAl-IMtCH and PAl-PMt(500)CH materials with respect to the initial pH change [28,29].

The parent clay exhibited a pH_PZC_ value of 7.8; after pillaring with the aluminium species, it decreased to 4.7. This decrease was related to the acidic nature of the pillaring species [66,67,68]. Meanwhile, the porous clay heterostructure (PAl-IMTCH) displaced a value of 6. The conventional porous clay heterostructures showed values between 4.7 and 5.7 [67,68]. These values depended on the inserted metal in the silica framework and the type of used clays [28,67,69]. The silica decorated pillared material (for example, the PAl-PMt(500)CH) has a pH_PZC_ value of 5.6. This value was higher than the pillared clay (Al-PMt(500)).

At pH levels lower than the proper pH_pzc_, it is evident that the adsorbent has a net positive charge. Strong coulombic repulsions between the dye and the adsorbent consequently occur. Repulsive forces, however, diminished at pH values greater than the adsorbent’s corresponding pH_pzc_ values [70]. As a result, more dye was removed because of the enhanced electrostatic attraction between the positively charged functional groups on BB-41 and the negatively charged surface of the PCH materials.

#### 3.7.3. Effect of BB-41 Initial Concentration on Elimination Effectiveness and on the Removal Capacity

**Figure 7:**
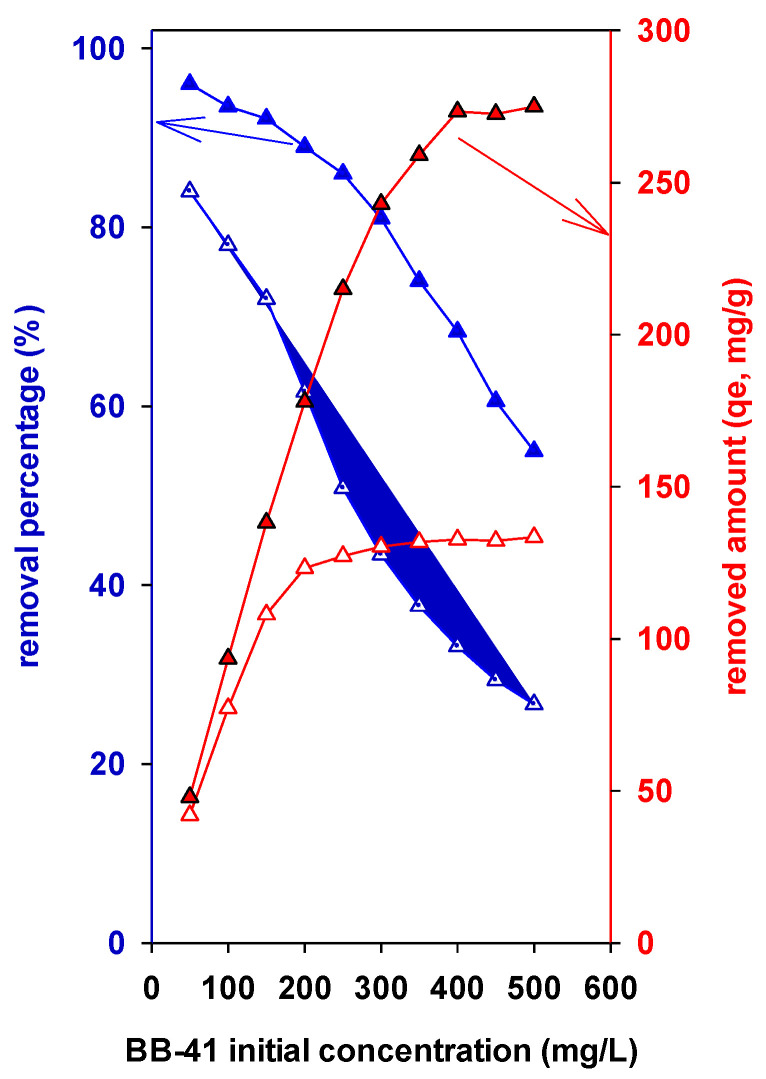
Effect of the BB-41 initial concentration of the removal properties of the PAl-IMtCH (filled triangles) and PAl-PMt(500)CH (non-filled triangles).

The effect of the concentration indicated that when the concentration of the BB-41 dye increases from 50 to 500 mg/L, the removal efficiency (the removed amount) improved as well (Figure 7). It was improved from 48 mg/g to 278 mg/g. This suggests that the BB-41 molecules migrate to the PAl-IMtCH’s surface because of the significant concentration differential between the two phases [71]. The removal process is improved as a result of the increased availability of surface sites on the PAl-IMtCH, facilitating greater dye removal. At low BB-41 concentrations, a strong removal effectiveness is established. This trend indicates that the transfer of mass among the water and solid stages is getting more challenging; ultimately, the adsorbate phase will run out of vacant sites [27,28,69]. Furthermore, incoming dyes are repelled by the adsorbate, since it already has adsorbate on the surface of the PAl-IMtCH. This results in a reduction in BB-41 effectiveness (removal percentage, %) at high concentrations, as opposed to low concentrations (Figure 7). The PAl-PMt(500)CH exhibited the same behavior. However, the removal efficiency and elimination effectiveness values were lower (Figure 7). This fact could be related to the structures and the surface areas of the two materials.

#### 3.7.4. Effect of Calcination Temperature of Al-IMt

The intercalated precursor was calcined at different temperatures from 300 °C to 800 °C before attempts to synthesize the PCHs. As already noted, the substantial interaction between the layers of clay and the pillared species prevented the successful achievement of the PCH materials.

The resulting materials’ removal capabilities turned out to be lower than that of the PCH from the Al-precursor (Al-IMt), but they were still higher than those of the original pillared clays, according to the removal tests. They were in the range from 105 mg to 160 mg/g. The presence of the silica phase resulting from the reaction of TEOS and C_12_amine may be the cause of the increase in removal efficiency. Attempts to improve the removal efficiency were adopted by treating the pillared 500 material (Al-PMt(500)) with an ultrasound bath (as reported above) to break down the bonding alumina pillars. The treatment of pillared clays after calcination with ultrasonic waves was not reported in the literature. However, the ultrasonic treatment was reported to synthesize pillared clay during the aging and intercalation steps. The PXRD indicated that the structure was not changed and no collapse of the pillars occurred. Thus, the synthesis of the PCH was not achieved, and slight variation in the BB-41 removal was noted (about 5%).

The contribution of the amorphous silica decorating the pillared materials was investigated. A mixture of C_12_amine and TEOS at the same conditions used to prepare the PCH without adding clay mineral, and calcined at 550 °C for 6 h, indicated that an amorphous silica phase resulted (PXRD data). The removal tests of basic blue 41 indicated that this phase has the capability to remove the BB-41 but the amount was limited to 25–30 mg/g.

#### 3.7.5. Modeling the Isotherm Data

Adsorption isotherms, which plot the equilibrium loading against the equilibrium concentration of an aqueous solution, show how adsorbate molecules interact with adsorbent particles and are therefore essential for maximizing the utilization of solid materials. The adsorption equilibrium is the subject of numerous theories, including the two-parameter models of Langmuir, Freundlich, and Redlich–Peterson [71,72,73]. In this study, the Langmuir model was adopted for comparison purposes related to previous studies. The data fit well to the Langmuir model, using linear equation. The parameters of Langmuir isotherms are reported in Table 4.

R^2^ values were close to 1 and confirm that removal on homogeneous surface sites occur. Pearson’s correlation coefficients are close to 1 and indicate a strong correlation between C_e_ and C_e_/q_e_ as presented by the linear equation of the Langmuir model. The maximum monolayer capacity (q_max_) of the BB-41 dye was achieved for the PCH material prepared from the Al_13_ intercalated precursor; it reached 274 mg/g. However, the PCH materials prepared from pillared clays calcined at different temperatures (300 °C to 800 °C) exhibited lower q_max_ values ranging from 76 to 160 mg/g. A selected pillared clay calcined at 500 °C (Al-PMt(500)) removed about 88 mg of BB-41/g. This value was higher than the parent clay mineral (57 mg/g) and confirmed the success of the pillaring process. The quantity depended on the calcination temperatures and a drop was observed at 800 °C due to the destruction of the clay layers and the collapse of the pillaring species.

All the dye-adsorbent systems under investigation had q_max_ parameter values that were higher than the experimental values, which could mean that the monolayer was not completely covered (Table 4). For BB-41, the computed value of the K_L_ parameter, which is associated with the dyes’ adsorption energy, fell between 0.225 and 0.054. This suggests that BB-41 has a higher affinity [73]. The removal of the BB-41 over PAl-PMt(700)CH resulted in the lowest Langmuir constant (K_L_), indicating a decreased affinity of the dye for the material surface.

The maximum adsorption capacity (q_max_) values (mg/g) were converted to maximum surface concentrations (Γ_max_, mg/m^2^) [28], which are presented in Table 4. Under real experimental conditions, the maximum surface concentration for the PAl-IMtCH material reached a maximum of 0.311 mg/m^2^. For the PAl-PMt(X)CH materials, the maximum surface concentration decreased in general as the temperature of calcination of pillared clay increased, and it reached a value of 0.371 mg/m^2^ for the PAl-PMt(300)CH material. According to three-dimensional computation, the BB-41 dye has a planar shape that is 1.716 nm in length, 0.665 nm in width, and 0.665 nm in thickness. These numbers closely matched those described in the literature [74]. The monolayer capacity of the BB-41 was calculated to be 0.618 mg/g based on its planar area. The findings showed that, in the current experimental setup, the q_max_ values for every material used did not surpass a monolayer of the BB-41 molecules, in good agreement with the theoretical values of q_max_ compared to the experimental ones as reported above. The adsorption of dye molecules could occur via the diffusion into the pores of used materials, as reported for other materials. The solute molecules diffuse in the pore structure of the adsorbents with a diameter of 1.3–1.8 times higher than their diameters [27]. Based on the dimensions of the BB-41 molecules and the pore of the Zr(X)-PCH materials, the BB-41 molecules diffuse easily in these pores.

The maximal removal capacity (q_max_) was compared with a few known sorbents based on aluminosilicates to evaluate the removal performance of the Al-PCHs (Table 5). The modification of Mt has enhanced the removal capacity from 55 mg/g to 88 mg/g for alumina pillared clay (500 °C). This value was lower compared to the zirconia pillared clay (500 °C) due to the difference of the specific surface area available for removal of the BB-41. The prepared PCH from the aluminium intercalated species exhibited an interesting removal efficiency of 274 mg/g; however, this was still lower than the PCH synthesized zirconium intercalated species. The latter removed 224 mg/g to 346 mg/g, as well as nanoporous silica material. The PCH prepared from pillared alumina clays exhibited a higher removal capacity of BB-41 than the starting pillared clays. The main difference between the reported materials could be related to the specific surface area and the pore size.

#### 3.7.6. Regeneration Performance

In addition to having a strong adsorption capacity, a possible adsorbent for dye removal needs to have a recovery capacity that is economically significant. Various methods for regenerating the adsorbents have been documented [81,82,83]. The parameters outlined in Section 2.5 were followed during the desorption trials to regenerate the sorbent for additional removal cycles.

The regeneration study was restricted to a sustainable friendly environment approach that avoided producing harmful chemicals [29].

**Figure 8:**
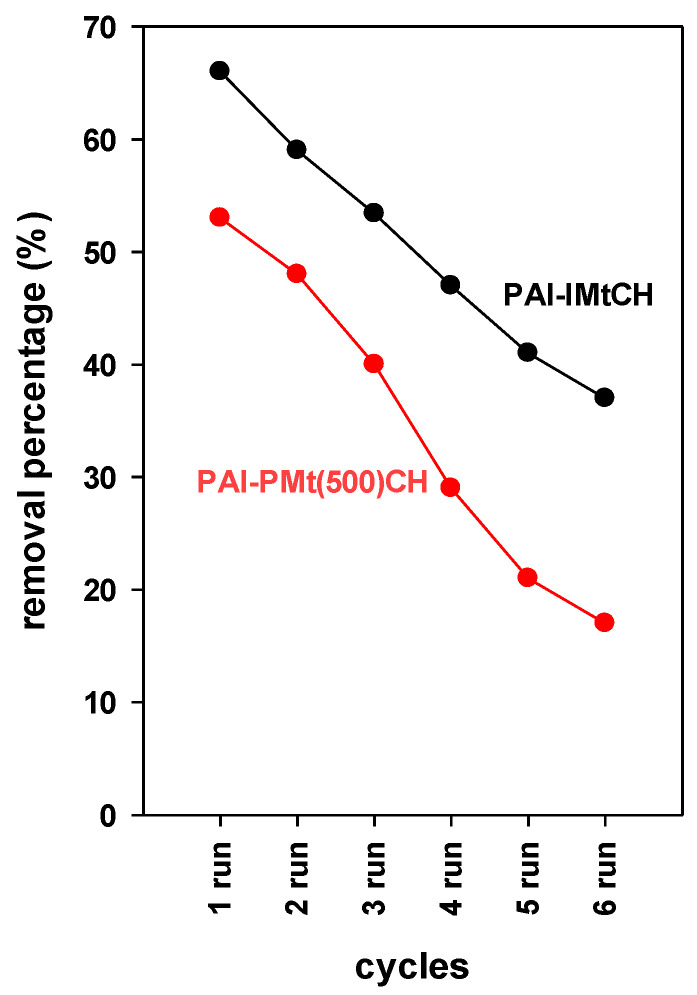
Variation of removal percentage (%) after different regeneration cycles.

Figure 8 displays the regeneration data of selected samples in six consecutive cycles of BB-41 elimination. After three cycles, the PAl-IMtCH material showed sustainable removal properties, according to the analysis, with a decline from the original value of 66% to 53%. After six cycles, this number dropped even further, reaching 37%. Overall, there was a 26% decrease in activity. According to the regeneration tests, at the sixth cycle, PAl-PMt(500)CH had lost roughly 36% of its initial activity. It requires two cycles for the initial efficiency to be sustained. Comparable data were stated for layered silicates and other PCH materials [27,28,64].

These results showed that PAl-IMtCH material was easier to break down the removed BB-41 dyes than PAl-PMt(500)CH. They also showed that the catalyst was easily accessible to the removed BB-41 in PAl-IMtCH pores, which was not the case in PAl-PMt(500)CH. Furthermore, Table 4 shows that PAl-IMtCH is more acidic than PAl-PMt(500)CH, which may cause the removed BB-41 molecules to be destroyed. However, because of the smaller pores and lower interlayer spacing of 1.75 nm in the resulting PCH from pillared material, it was challenging to reach the removed BB-41 molecules. An additional explanation might be that the eliminated BB-41 molecules in PAl-PMt(500)CH are firmly bonded to the removal sites [27,28].

#### 3.7.7. Single-Stage Batch Design Process

To translate the results of the laboratory bench-scale study to a large-scale study, which may then be utilized for developing an industrial wastewater treatment system, a batch adsorber model must be developed [84,85]. The objective is to estimate the quantity of materials needed to eliminate a particular percentage of the pollutant from fixed amounts of polluted water with a known concentration. Assume the volume V (L) of the BB-41 solution and the concentration that decreases from C_o_ to C_1_ (mg/L). The loading shifts from q_o_ to q_1_, and the adsorbent dosage is M (mg). At first, q_o_ = 0, and over time, the mass balance represents the amount of BB-41 removed from the solution. The formula for calculating the mass balance is as follows [86]:

V(*C_o_* − *C*_1_) = M(*q_o_* − *q*_1_) (1)

q_1_ approaches q_e_ and C_1_ approaches *C_e_* at equilibrium. The removal data fit well the Langmuir isotherm, therefore the single stage adsorber equation is as follows [27]:

(2) $$\frac{m}{V}=\frac{C_{o}-C_{e}}{q_{e}}=\frac{C_{o}-C_{e}}{\frac{q_{m}K_{L}C_{e}}{1+K_{L}C_{e}}}$$

Replacing *C_o_* − *C_e_* and *C_e_* expressions by *C_o_* and removal percentage (*R*%) Equation (2) could be rewritten as

(3) $$\frac{m}{V}=\frac{C_{o}-C_{e}}{q_{e}}=\frac{RC_{o}}{q_{m}\frac{K_{L}(1-R)C_{o}}{1+K_{L}(1-R)C_{o}}}$$

**Figure 9:**
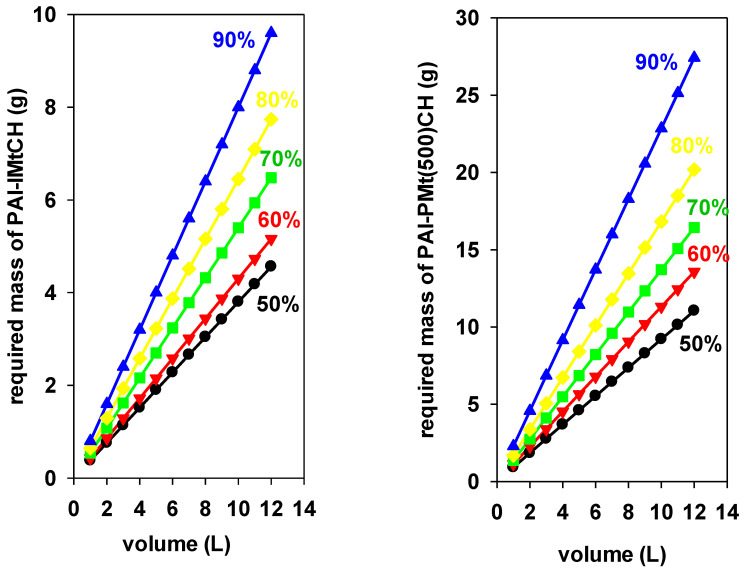
Required masses of PAl-IMtCH (**left**) and PAl-PMt(500)CH (**right**) to reduce different volumes (L) of BB-41 solutions (C_i_ = 200 mg/L) to different removal percentages.

The masses of PAl-PMtCH and PAl-PMT(500)CH necessary to remove various volumes of BB-41 with C_i_ of 200 mg/L at varied removal percentages (50 to 90%, with a 10% increment) are shown in Figure 9. Two essential tendencies were noted as follows: more solids’ masses are required as the removal percentages (R%) increased for a particular volume of treated solution; and, as the volume of solution being treated increased, the predicted amounts of solids also increased [28] for a fixed removal percentage. The plot could be used to calculate the needed mass of the solid to remove BB-41 from a specified volume and concentration of the solution. The necessary mass of PAl-IMtCH to reduce the starting concentration from 200 mg/L to 100, 140, and 180 mg/L if 10 L of BB-41 solution needs to be treated is 3.8, 5.4, and 8 g, respectively.

The masses of PAl-PMt(500)CH required to treat the same volume of dye solution under the same conditions are 9.2, 6.8, and 11.4 g, respectively. The greater removal capacity (q_max_) of PAl-IMtCH (274 mg/g) as opposed to Pal-PMt(500)CH (120 mg/g) was the cause of the significant difference. Comparable results have been published for layered silicates and other porous clay heterostructures [27,28,64,65]. Figure 9 can be extended to include other values of pesticides removals till 100% and can be modifies to include any other conditions from initial concentration to solution temperature.

## 4. Conclusions

Using an intercalated Al_13_ montmorillonite precursor and their pillared derivatives (obtained after calcining the precursor from 300 °C to 800 °C), an attempt was made to synthesize porous clay heterostructures (PCHs). The PCH material was successfully synthesized from the intercalated aluminium precursor with exceptional properties such as a high surface area of 880 m^2^/g and an acidity value of 0.60 moles of H^+^/g. The direct insertion of the Al species into the silica framework was achieved and enhanced the physico-chemical properties. Unlike the intercalated precursor, the pillared alumina montmorillonites did not lead to the synthesis of the PCH materials. During calcination, the intercalated Al_13_ species were converted to aluminium oxide and they were strongly bonded to the clay mineral sheets, which made them difficult to be exchanged with the C_12_amine co-surfactant. In this case, the silica phase could decorate the external surface of the pillared clays with blockage of the pores. The resulting materials were used to eliminate the BB-41. At pH 8, the elimination was most successful. Compared to the PAl-PMt(X)CHs, the Pal-CH performed better with a maximum removed amount of 274 mg/g. The greater surface area, porosity, and acidity may be the cause of this. Most of the experimental data fit a monolayer isotherm of the Langmuir type. Following three or four consecutive regeneration cycles, the materials under study underwent regeneration. Of the initial 200 mg/L, 90% of the concentration may be eliminated with a lower amount of PAl-IMtCH (8 g) for a treated volume of 10 L, according to modelling of the equilibrium data and the batch single stage design.

## Figures and Tables

**Table 1:** XRF data of the parent clay and its pillared counterparts.

Samples	Mt	Al-IMt	Al-PMt(300)	Al-PMt(400)	Al-PMt(500)	Al-PMt(600)	Al-PMt(700)	Al-PMt(800)
SiO_2_	56	(79.9) *	57.9 (63.3)	56.4 (63.9)	57.2 (62.2)	57.7 (61.1)	57.7 (61.1)	58.8 (61.9)
Al_2_O_3_	22.4	22.2 (5.54)	23.5 (17.9)	22.8 (16.3)	23.8 (18.7)	23.3 (17.7)	23.1 (18.6)	24.1 (19.2)
MgO	3.00	2.23 (0.66)	2.27 (1.68)	2.21 (1.59)	2.28 (1.72)	2.26 (1.68)	2.28 (1.79)	2.38 (1.87)
CaO	1.77	0.05 (0.014)	0.049 (0.042)	0.055 (0.033)	0.047 (0.026)	0.047 (0.052)	0.653 (0.523)	0.692 (0.547)
Fe_2_O_3_	0.884	0.669 (0.139)	0.0663 (0.435)	0.628 (0.467)	0.063 (0.528)	0.643 (0.506)	0.046 (0.044)	0.040 (0.036)
TiO_2_	0.236	0.019 (0.03)	0.213 (0.144)	0.224 (0.164)	0.204 (0.164)	0.204 (0.164)	0.206 (0.151)	0.223 (0.169)
K_2_O	0.108	0.062 (0.03)	0.096 (0.054)	0.084 (0.055)	0.072 (0.062)	0.088 (0.062)	0.079 (0.069)	0.066 (0.062)

* Values between brackets correspond to the resulting materials after reaction with C_12_amine and TEOS.

**Table 2:** Microtextural properties of the Al-IMt precursor calcined at different temperatures, and their PCH counterparts.

Samples	S_BET_ (m^2^/g)	V_µp_ (cm^3^/g)	S_ext_ (m^2^/g)	T.P.V (cc/g)	A.P.D (nm)
Mt	90	0.0	95	0.051	5.8
Al-IMt	880 (318)	0.0 (0.098)	880 (134)	0.851 (0.257)	3.82 (3.23)
Al-PMt(300)	383 (269)	0.024(0.078)	335 (117)	0.310 (0.216)	3.22 (3.21)
Al-PMt(400)	453 (285)	0.024 (0.087)	400 (121)	0.389 (0.242)	3.43 (3.34)
Al-PMt(500)	360 (281)	0.043 (0.086)	275 (160)	0.239 (0.237)	2.65 (3.37)
Al-PMt(600)	380 (227)	0.020 (0.06)	329 (113)	0.245 (0.239)	2.63 (3.58)
Al-PM(700)	340 (165)	0.01 (0.037)	303 (94)	0.170 (0.21)	2.48 (4.1)
Al-PMt(800)	244 (30)	0.0 (0)	244 (30)	0.157 (0.075)	2.57 (9.9)

V_µp_: volume micropore; T.P.V: total pore volume; A.P.D.: average pore diameter. The values between brackets correspond to the starting materials.

**Table 3:** Acidity concentration of the Al-IMt precursor calcined at different temperatures and their PCH counter parts deduced form desorption of cyclohexylamine probe molecule.

Samples	Acidity *	Samples	Acidity *
Mt(500)	0.51	---	---
Al-IMt	0.32	PAl-IMtCH	0.60
Al-PMt(300)	0.71	PAl-PMt(300)CH	0.73
Al-PMt(400)	0.58	PAl-PMt(400)CH	0.62
Al-PMt(500)	0.49	PAl-PMt(500)CH	0.58
Al-PMt(600)	0.47	PAl-PMt(600)CH	0.59
Al-PMt(700)	0.34	PAl-PMt(700)CH	0.59
Al-PMt(800)	0.29	PAl-PMt(800)CH	0.59

* mmoles of H^+^/g of sample.

**Table 4:** Langmuir model parameters for removal of BB-41 by different materials.

Samples	q_max_ (mg/g)	K_L_ (g/L)	R^2^	PCC *	Γ_max_, mg/m^2^
Mt	57	0.0289	0.9854	0.9993	0.633
Al-PMt(500)	88	0.0564	0.9999	0.9999	0.313
PAl-IMtCH	274	0.2280	0.9996	0.9997	0.311
PAl-PMt(300)CH	142	0.1635	0.9998	0.9999	0.371
PAl-PMt(400)CH	160	0.1743	0.9998	0.9998	0.353
PAl-PMt(500)CH	120	0.0955	0.9996	0.9999	0.316
PAl-PMt(600)CH	140	0.1354	0.9998	0.9999	0.369
PAl-PM(700)CH	105	0.0787	0.9997	0.9999	0.309
PAl-PM(800)CH	76	0.054.	0.9996	0.9997	0.311

* PCC corresponds to Pearson’s correlation coefficient.

**Table 5:** Maximum removal capacities of some selected materials.

Samples	q_max_ (mg/g)	Reference
Montmorillonite (Mt)	55 mg/g	[28]
Saudi Local clays	50–70	[75]
Brick wastes	60–70	[76]
Natural zeolite	60–70	[74]
Sodalite Zeolite	39	[77]
Mn modified diatomite	77	[78]
Bentonite *	173	[79]
Nanoporous silica	345	[80]
Zirconia pillared clay (500 °C)^+^	114	[27]
Al-PCH	274	[28]
Zr-PCH	224–346	[27]
Alumina pillared clays (500 °C)^+^	88	This study
PCH from pillared clays	100–165	This study

* Bentonite-poly(p-hydroxybenzoic acid) composite. (500 °C)+ indicates the calcination temperature.

## Data Availability

The original contributions presented in the study are included in the article, further inquiries can be directed to the corresponding authors.
